# Transcriptomic Evidence Identifies Two TMBIM Subgroups with Opposing Prognostic Associations in Glioma

**DOI:** 10.3390/biology15141179

**Published:** 2026-07-17

**Authors:** Sofia Ramos, Gonçalo Pereira, Marta Martins, Ana Sofia Fernandes, Nuno Saraiva

**Affiliations:** 1 CBIOS, ECTS, Lusófona University, Campo Grande 376, 1749-024 Lisbon, Portugal; sofia.ramos@ulusofona.pt (S.R.); fc64497@alunos.ciencias.ulisboa.pt (G.P.); martafilipa31@gmail.com (M.M.); ana.fernandes@ulusofona.pt (A.S.F.); 2 Department of Biology, Faculty of Sciences, University of Lisbon, 1749-016 Lisbon, Portugal; 3 Gulbenkian Institute for Molecular Medicine (GIMM), Edifício Egas Moniz, Avenida Professor Egas Moniz, 1649-028 Lisboa, Portugal; 4 Department of Biomedical Sciences, University of Alcalá, Ctra. Madrid-Barcelona Km. 33.600, 28871 Alcalá de Henares, Madrid, Spain

**Keywords:** glioma, glioblastoma, TMBIM, therapeutic target selectivity, prognosis biomarkers, biological processes

## Abstract

Gliomas are the most common and aggressive primary brain tumours, with glioblastoma (GB) exhibiting a poor prognosis and limited therapeutic response. Dysregulation of intracellular ion homeostasis, particularly Ca^2+^ signalling, has emerged as a key contributor to glioma progression. The transmembrane BAX inhibitor motif-containing (TMBIM) protein family regulates intracellular Ca^2+^ flux and cell survival; however, their role in glioma remains incompletely understood. Gene expression and clinical data from TCGA, CGGA, and Rembrandt cohorts were analysed to assess the association between TMBIM1-6 expression, tumour grade, and patient survival. Correlation analyses identified TMBIM-associated gene networks, followed by functional enrichment to characterise underlying biological processes and molecular functions. The TMBIM family members segregated into two distinct groups with opposing clinical associations. TMBIM1, TMBIM4, and TMBIM6 were upregulated and associated with poor survival, whereas TMBIM2, TMBIM3, and TMBIM5 were downregulated and associated with increased survival. Functional enrichment analyses revealed two conserved gene expression programmes: TMBIM1/4/6 are linked to membrane trafficking, metabolic and bioenergetic adaptation, while TMBIM2/3/5 are associated with cell cycle regulation and chromosomal instability. These findings uncover a previously unrecognised functional divergence within the TMBIM family in glioma. This bipartite organisation highlights TMBIM proteins as potential prognostic markers and suggests that selective targeting of specific TMBIM subgroups may improve therapeutic strategies.

## Simple Summary

Gliomas are the most common and aggressive brain tumours, and current treatments are often not effective, especially in the most severe cases. One reason for this is that tumour cells can adapt and survive under stress, partly by altering the movement of calcium ions inside the cell. In this study, we investigated a group of genes (the members of the transmembrane BAX inhibitor motif-containing (TMBIM) family) that regulate calcium ion movement and that help cells to survive and invade. By analysing data from hundreds of glioma patients, we found that these genes fall into two distinct groups with opposite effects. One group is linked to worse patient survival, while the other is associated with better outcomes. We also discovered that these two groups are associated with different biological processes, such as cell growth, cell division, invasion, and tumour cell interaction with their environment. These findings improve our understanding of how gliomas develop and progress. Importantly, the data we generated can guide future treatment design to target TMBIM proteins specifically, rather than the entire family, leading to more effective and less toxic therapies for patients.

## 1. Introduction

Gliomas are the most common and therapeutically challenging primary brain tumours, characterised by high heterogeneity and poor patient outcome, particularly in high-grade subtypes such as glioblastoma (GB). Gliomas are classified into four grades according to tumour aggressiveness, histology, and molecular genetic markers. Grade IV corresponds to GB and is the most severe form of all gliomas [1]. Although representing approximately 30% of all primary brain tumours, gliomas account for 80% of malignant brain tumours and are the leading cause of brain tumour-related mortality [2]. Despite recent advancements in cancer therapies, the prognosis and patient survival remain limited due to intrinsic tumour hallmarks and the presence of the blood–brain barrier, which restricts the delivery of blood-circulating drugs to the brain [3,4]. This highlights the urgent need for a deeper understanding of the molecular mechanisms driving glioma progression, as well as their resistance to therapy and frequent recurrence [5].

Glioma progression involves complex regulatory networks controlling cell proliferation, apoptosis, invasion, and metabolic reprogramming [6]. Emerging hallmarks of glioma biology, such as the dysregulation of intracellular trafficking, ion fluxes, and the secretory pathways, influence tumour cell survival, proliferation, and invasion [7]. These processes are tightly linked to ionic homeostasis, especially to calcium ion (Ca^2+^) signalling, which acts as a second messenger that controls cell cycle progression, cytoskeletal dynamics, and responses to cellular stress, and plays a central role in regulating cell fate decisions [8]. Alterations in intracellular Ca^2+^ homeostasis are often driven by abnormal activity of channels in the plasma membrane and in intracellular organelles such as the endoplasmic reticulum (ER), the Golgi apparatus (GA), secretory pathway vesicles, and mitochondria [9,10]. Thus, Ca^2+^ channels are potential key modulators of glioma progression and may represent promising therapy targets.

Members of the transmembrane BAX inhibitor motif-containing (TMBIM) protein family have been reported to modulate intracellular Ca^2+^ and cell survival [11,12,13]. The TMBIM family contains six evolutionarily conserved channels (TMBIM1-6). These proteins modulate intracellular Ca^2+^ fluxes across the membranes of intracellular organelles and also influence ion fluxes between intracellular and extracellular spaces [14]. TMBIM proteins have distinct subcellular localisations, including the ER (TMBIM2-3 and TMBIM6), GA (TMBIM1-4), mitochondria (TMBIM5), lysosomes and endosomes (TMBIM1), and even the plasma membrane (TMBIM2), which are associated with their roles in maintaining cellular homeostasis [15,16,17,18,19,20]. This localisation can vary in some cases according to expression level or cell type. Through their influence on Ca^2+^ fluxes, mitochondrial metabolic status, ER stress responses, autophagy, cell motility, and apoptosis, TMBIM proteins have been implicated in cancer progression [11,14,21,22,23,24,25,26,27]. However, a comprehensive understanding of the diverse roles that each TMBIM protein plays in glioma remains lacking, particularly regarding their association with tumour grade, patient survival, and overall molecular mechanisms.

In this study, we characterise the expression profile of TMBIM1-6 in glioma and evaluate its association with tumour grade, patient survival, and cellular mechanisms relevant to tumour progression. Here we describe for the first time that TMBIM1, 4, and 6 form a distinct cluster from TMBIM2, 3, and 5, showing opposite correlations with tumour grade, patient survival, and gene expression. Overall, these findings highlight a previously unrecognised functional divergence within the TMBIM family. This is particularly important to guide future development of selective therapeutic strategies involving specific TMBIM inhibition.

## 2. Materials and Methods

### 2.1. Patient and Gene Expression Data

Patient-level genomic and clinical data were obtained from The Cancer Genome Atlas-Glioblastoma and Low-Grade Glioma (TCGA-GBMLGG) [28,29], and Rembrandt [30] cohorts, accessed through the GlioVis platform (https://gliovis.bioinfo.cnio.es/, accessed on 15 November 2025) [31]. The TCGA-GBMLGG cohort comprises 667 patients (152 GB and 515 LGG) with gene expression profiles generated by RNA sequencing, and the Rembrandt cohort comprises 444 patients (219 GB and 225 LGG) with gene expression profiles measured by microarray technology. The mRNA microarray dataset from the Chinese Glioma Genome Atlas (CGGA; https://www.cgga.org.cn/index.jsp, accessed on 15 November 2025), with 301 patients (174 GB, 124 LGG, and 2 undefined), was also included [32].

### 2.2. Association Between TMBIMs Expression and Glioma Tumour Grade

The association between TMBIM family gene expression and glioma tumour grade (II-IV) and Isocitrate Dehydrogenase 1 (IDH) status were assessed through an initial descriptive analysis, followed by a one-way ANOVA test to determine whether statistically significant differences existed among the groups. Statistical analyses were performed in Python v3.12 using the Google Colaboratory (Google Colab) platform. Data handling and pre-processing were conducted using Pandas (v.2.2.2). Numerical computations were performed using NumPy (v.2.0.2). Analysis of variance (ANOVA) was performed using SciPy (v.1.16.3). When the ANOVA indicated significant effects (*p* < 0.05), Tukey’s Honest Significant Difference (HSD) post hoc test was applied to identify which group means differed from each other. Statistical significance was evaluated at thresholds of *p* < 0.001, *p* < 0.01, and *p* < 0.05. Graphics were generated with GraphPad Prism10.1.1.

### 2.3. Association Between TMBIM Expression and Glioma Patient Survival

To evaluate the association between the expression of TMBIM family genes and the overall survival (OS) of glioma patients, Kaplan–Meier survival analyses and Cox proportional hazards regression models were performed in Python v.3.12 using the Google Colab environment. Data processing and management were carried out using Pandas (v.2.2.2), while numerical computations were performed with NumPy (v.2.0.2). Survival analyses, including Kaplan–Meier curve estimation, log-rank tests, and Cox proportional hazards modelling, were conducted using the Lifelines package (v.0.30.1). For each gene, patients were dichotomised into high- and low-expression groups using the median gene expression as the cut-off within each dataset. Hazard ratios (HRs) and 95% confidence intervals (CIs) were derived from the Cox models. To integrate results across cohorts, a weighted median of HRs was calculated, accompanied by corresponding upper and lower limits derived from the distribution of the cohort-specific HRs, providing a robust, variance-adjusted summary estimate of the prognostic effect. Graphics were generated with GraphPad Prism10.1.1.

### 2.4. Identification of Genes with Expression Correlation with TMBIM1-6 in Glioma

To characterise gene expression correlation patterns between TMBIM genes and the related transcriptome, genes exhibiting the strongest expression correlations with each TMBIM gene were identified using the Pearson correlation coefficient (PCC) in the TCGA-GBMLGG and Rembrandt datasets. For each TMBIM gene, the top 300 genes with the highest PCC were identified. An absolute correlation threshold of PCC > 0.4 or <−0.4 was also applied. The same analytical framework was subsequently applied in a subtype-specific manner, using TCGA datasets, in which LGG and GBM samples were analysed independently: TCGA-GBM (GB, grade IV glioma) and TCGA-LGG (grade II and III glioma) classified according to the TCGA Research Network [29]. In this context, the 100 genes with higher PCC with each TMBIM were identified in LGG and GB. For each group of identified genes for each TMBIM, the expression dysregulation trend (for those with PCC > 0.4 or <−0.4) was calculated for the other TMBIMs. The percentage of shared and oppositely correlated genes was quantified to evaluate the similarity and disparity of correlation patterns across TMBIM members in the TCGA-GBMLGG and Rembrandt datasets, as well as between LGG and GB. In addition, pairwise expression correlations among TMBIM family members were calculated in both datasets and within each glioma subtype. Statistical analyses were performed in Python v3.12 using the Google Colab platform. Gene expression correlation analyses were conducted using Pandas (v.2.2.2) and NumPy (v2.0.2), with the Pearson correlation coefficient computed using SciPy (v.1.16.3). Heatmaps were generated with GraphPad Prism10.1.1. Tables containing PCC values and gene names obtained in this analysis are available at https://doi.org/10.5281/zenodo.19226542.

### 2.5. Gene Ontology Analysis of Correlated Genes

To detect shared gene expression correlation patterns between TMBIMs, the identified TCGA-GBMLGG gene sets were intersected using Venn diagram analysis. Venn diagram gene lists were deposited at https://doi.org/10.5281/zenodo.19226542. These overlapping gene sets underwent functional enrichment analysis. Gene Ontology Biological Process (GO-BP) and Molecular Function (GO-MF) enrichment analyses were performed using the clusterProfiler package (v4.14.3) [33] from BiocManager v3.20 in RStudio (v4.4.0) for all pairs of TMBIMs. Enriched terms were filtered using a false discovery rate (FDR) < 0.05, ranked by fold enrichment, and the top ten terms within each ontology category were selected. Enrichment analysis was visualised using Sankey plots from SRplot [34] when the number of genes was sufficient to produce statistically significant GO enrichment after multiple-testing correction.

## 3. Results and Discussion

### 3.1. Opposing Glioma Patient Survival Associations Among the TMBIM Family

Since the TMBIM family regulates intracellular ion flux and several cellular processes associated with key features of glioma progression, such as cell invasion, proliferation and metabolic reprogramming, we investigated the possible association of these genes with glioma patient outcome. To address this, an association between TMBIM gene expression and overall survival in glioma patients was assessed, using a median expression cut-off to stratify high- and low-expression groups.

**Figure 1:**
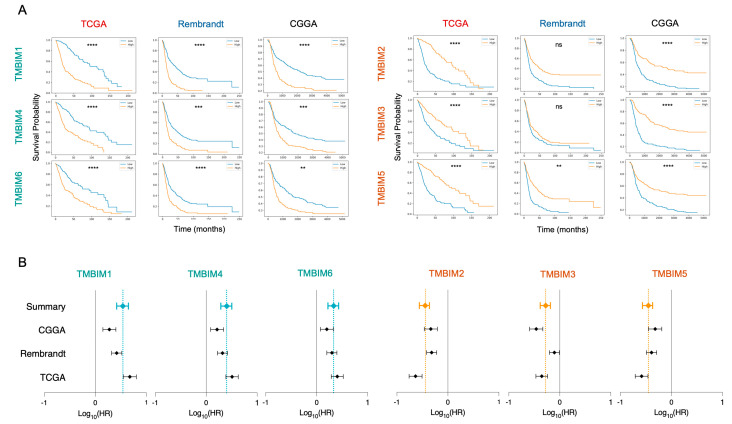
Association between TMBIM1-6 gene expression (mRNA) and glioma patient overall survival. (**A**) Overall survival was evaluated for TMBIM genes using Kaplan–Meier survival analyses and Cox proportional hazards regression models in the TCGA, Rembrandt, and CGGA datasets. Patients were stratified into high- and low-expression groups based on the median expression value within each dataset. Statistically significant differences between high and low expression group curves were assessed by a LogRank test ** *p* < 0.01, *** *p* < 0.001, **** *p* < 0.0001, ns non-significant. (**B**) Hazard ratios (HRs) and 95% confidence intervals (CIs) were estimated independently for each dataset, and a weighted median log10 (HR) was computed to summarise the overall effect across cohorts. Summary results were presented on a logarithmic scale, and the pooled estimate is highlighted as an integrated measure of association.

Kaplan–Meier survival analyses (Figure 1A) revealed a divergent survival pattern across TMBIM family members. Higher expression of TMBIM1, TMBIM4, and TMBIM6 was significantly associated with decreased survival, whereas elevated expression of TMBIM2, TMBIM3, and TMBIM5 correlated with increased overall survival. The same pattern was observed in the TCGA and CGGA cohorts. In the Rembrandt dataset, the same directional trend was detected; however, the associations for TMBIM2 and TMBIM3 did not reach statistical significance (*p* > 0.05). Cox proportional hazards analysis (Figure 1B) confirmed these findings, with TMBIM1, TMBIM4, and TMBIM6 exhibiting HR > 1, while TMBIM2, TMBIM3, and TMBIM5 showed HR < 1. These associations, consistently observed across three independent datasets, support the robustness of the prognostic stratification.

### 3.2. TMBIM Family Members Exhibit Divergent Expression Trends During Glioma Progression

**Figure 2:**
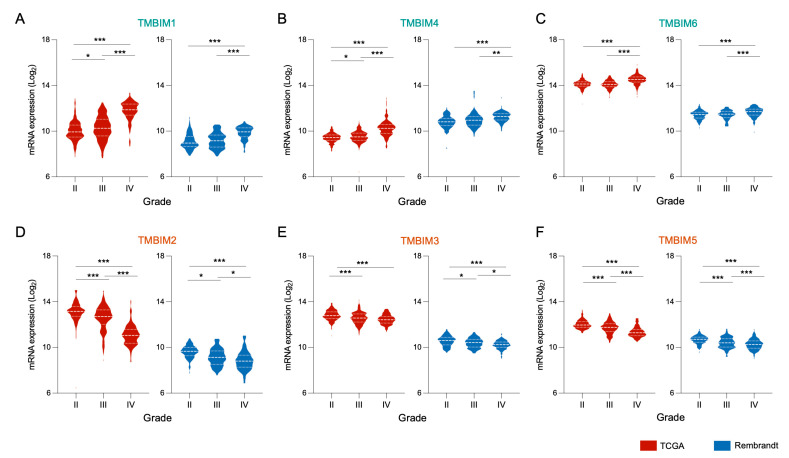
Association between TMBIM gene expression and glioma grade. (**A**) TMBIM1, (**B**) TMBIM4, (**C**) TMBIM6, (**D**) TMBIM2, (**E**) TMBIM3, and (**F**) TMBIM5 gene expression in glioma according to tumour grade (II, III, and IV) obtained from the TCGA and Rembrandt datasets. Group differences were assessed using one-way ANOVA followed by Tukey’s HSD post hoc test for pairwise comparisons. * *p* < 0.05; ** *p* < 0.01; *** *p* < 0.001.

To investigate the relationship between TMBIM family expression and tumour progression, gene expression across glioma grades II to IV was analysed. TMBIM family members exhibited two distinct and opposing grade-associated expression patterns (TCGA and Rembrandt datasets). TMBIM1, TMBIM4, and TMBIM6 demonstrated a progressive increase in expression with advancing tumour grade (Figure 2A–C), whereas TMBIM2, TMBIM3, and TMBIM5 showed a gradual decrease from grade II to grade IV (Figure 2D–F). These grade-dependent expression changes between grade II and grade IV were statistically significant in both datasets (*p* < 0.001), supporting a consistent association between TMBIM transcriptional profiles and glioma malignancy progression. The new adult-type diffuse glioma classification (included in the WHO Classification of Tumours of the Central Nervous System, 5th Edition (WHO CNS5) [35]) classifies the most aggressive type of glioma (glioblastoma, grade IV) based on IDH status (wild type (wt) or mutant). Considering this, the expression of TMBIMs’ association with the IDH status was analysed (Figure S1). The data obtained support a higher expression of TMBIM1, TMBIM4 and TMBIM6 in IDHwt gliomas and a higher expression of TMBIM2, TMBIM3 and TMBIM5 in IDHmutant gliomas. Importantly, the directionality of grade- and IDH-status-dependent expression changes was consistent with the observed survival associations (Figure 1 and Figure S1), further supporting the prognostic relevance of TMBIM family members in glioma.

### 3.3. TMBIM Transcriptionally Associated Genes Define Two Distinct Landscapes

**Figure 3:**
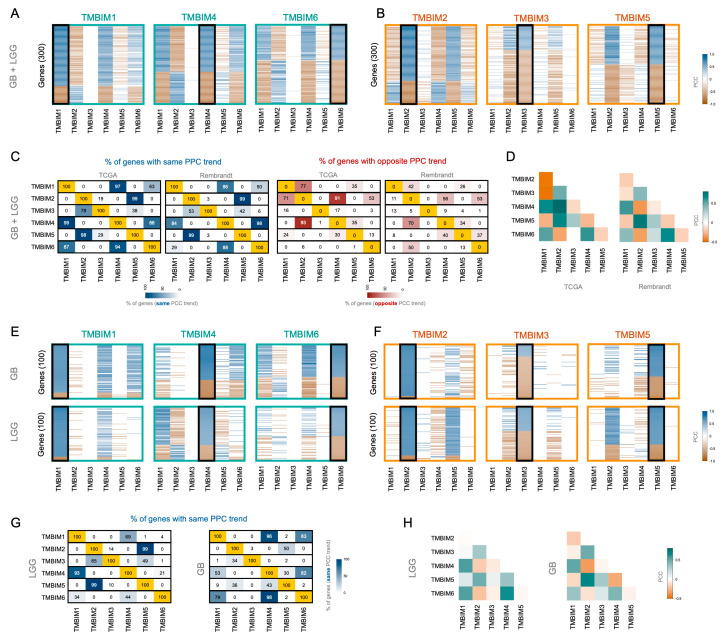
Gene expression correlation landscape of the TMBIM family in glioma. (**A**,**B**) Heatmaps displaying the correlation coefficient (PCC) of the top 300 genes most strongly correlated with each TMBIM family member (TCGA-GBMLGG dataset) for all six TMBIMs. The Pearson PCC based on TCGA-GBMLGG expression data is shown. Black boxes represent the TMBIM for which the most significant expression-correlated genes were obtained. These were analysed for their expression correlation with the remaining 5 TMBIMs. Only genes with PCC > 0.4 or >−0.4 are shown. (**C**) Percentage of genes with the same or opposite correlation trend (positive/negative) of the top 300 identified for each TMBIM from the two datasets (horizontal lines correspond to each set of 300 genes identified for each TMBIM). (**D**) Expression correlation between TMBIMs in glioma (all grades) from TCGA and Rembrandt datasets. (**E**,**F**) Subtype-specific correlation landscape of TMBIM family gene expression in TCGA glioma datasets. Heatmaps displaying the top 100 genes most strongly correlated with the expression of each TMBIM family member in LGG and GB samples. (**G**) Percentage of genes correlated with the top 100 identified for each TMBIM in LGG or GB separately. (**H**) Expression correlation between TMBIMs in LGG and GB from the TCGA dataset.

To investigate the relationship between each TMBIM family member and its associated transcriptional landscape in glioma, the 300 genes with the highest expression correlation coefficient were identified for each TMBIM using the TCGA (Figure 3A,B) and the Rembrandt datasets (Figure S2). For each TMBIM, the expression correlations of these 300 genes with the remaining five TMBIM family members were evaluated. A high level of overlap was observed between genes highly correlated with TMBIM1, TMBIM4, and TMBIM6, displaying a similar correlation pattern within this subgroup (Figure 3A). Quantification of co-dysregulated genes revealed a substantial percentage of overlap among their respective gene sets (Figure 3C). In contrast, genes associated with TMBIM2, TMBIM3, and TMBIM5 exhibited the opposite correlation profile (Figure 3B,C). Interestingly, a high percentage of TMBIM2 co-dysregulated genes show opposite trends of dysregulation (positive vs. negative) with TMBIM4 and TMBIM6 (Figure 3C).

Consistently, direct correlation analysis among TMBIM family members revealed strong positive correlations between TMBIM1, 4, and 6, whereas these genes showed negative correlations with TMBIM2, 3, and 5 (Figure 3D). Importantly, this bipartite correlation pattern was reproducible across both the TCGA and Rembrandt datasets.

Performing a separate correlation analysis for LGG and GB TCGA datasets, the opposite correlation pattern among TMBIM-associated gene sets was preserved (Figure 3E,F). However, the strength of these relationships differed between tumour subtypes. In GB, the shared transcriptional landscape was more pronounced among TMBIM1, 4, and 6, whereas the correlations involving genes associated with TMBIM2, 3, and 5 appeared more diffuse. Conversely, in LGG, stronger correlation structures were observed for gene sets associated with TMBIM4, 2, 3, and 5, whereas the transcriptional associations of TMBIM1 and TMBIM6 were more prominent in GB. These differences in correlation landscapes may reflect the well-established transcriptional divergence between LGG and GB [36,37]. Despite these variations, LGG and GB exhibited substantial overlap in genes with concordant correlation trends across TMBIM members (Figure 3G), aligned with a consistent correlation relationship among TMBIM genes (Figure 3H). An exception was observed for TMBIM3, which displayed comparatively weaker correlations, and the associations with genes correlated to TMBIM2 and TMBIM5 were modest, particularly in GB. Collectively, these findings indicate that the core TMBIM transcriptional network is largely preserved across glioma grades, while its relative strength and organisation vary between LGG and GB. The preservation of these transcriptional relationships across LGG and GB suggests that TMBIM-associated cellular processes are associated with glioma transcriptional reorganisation during tumour progression.

### 3.4. Functional Gene Enrichment Supports Two TMBIM-Associated Transcriptional Programmes

**Figure 4:**
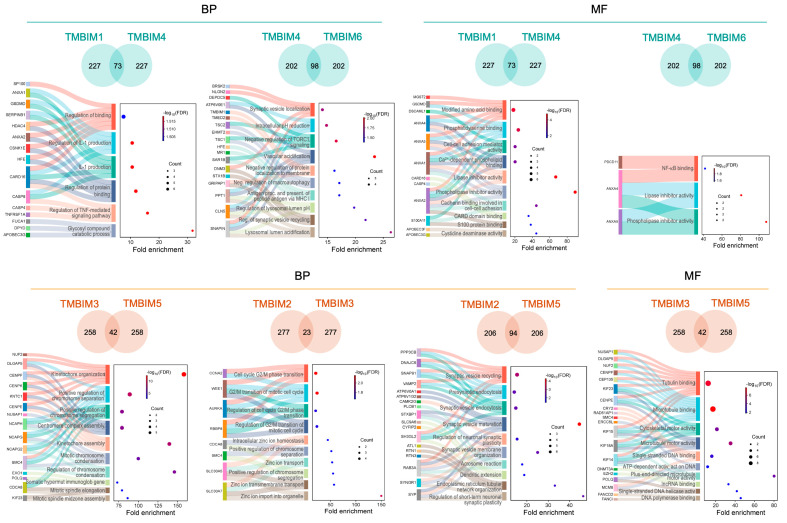
Biological processes (BPs) and molecular functions (MFs) associated with TMBIM1-6 co-dysregulated genes. Genes whose expression levels were simultaneously significantly correlated with two TMBIMs were analysed using Venn diagrams. Gene Ontology enrichment analysis (GOEA) was performed for each gene set with a sufficient number of overlapping genes, displaying the ten most significantly enriched biological processes. Pathways were selected based on False Discovery rate (FDR < 0.05) and ranked by fold enrichment.

To investigate the cellular processes linked to TMBIM-correlated genes, a functional enrichment analysis was conducted using genes whose expression patterns correlated with different TMBIM family members (Figure 4). Genes correlated with TMBIM1 and TMBIM4 were significantly enriched in glycan degradation and regulation of the TNF-mediated signalling pathway. Aberrant glycosylation promotes the accumulation of truncated glycans on glycoproteins and glycolipids, contributing to glioma aggressiveness, tumour progression, and therapeutic resistance [38]. In addition, several genes involved in TNF-mediated signalling, including Caspase Recruitment Domain Family Member 16 (CARD16), Caspase 8 (CASP8), and Caspase 4 (CASP4), are associated with apoptosis dysregulation and immune cell infiltration [39,40,41]. Consistently, TMBIM1 and TMBIM4 have also been implicated in the modulation of apoptosis and cell migration [21,24,25]. Notably, interleukin-1 (IL-1) signalling plays a pivotal role in glioma cell proliferation, invasion, and resistance to therapy through various mechanisms. The activation of IL-1β leads to the stimulation of oncogenic pathways, particularly the nuclear factor kappa-light-chain-enhancer of activated B cells (NF-κB) signalling cascade [42,43,44,45,46].

Genes correlated with TMBIM4 and TMBIM6 were associated with lysosomal and vascular acidification and synaptic vesicle localisation, recycling, and autophagy. These observations are compatible with the localisation of TMBIM4 and TMBIM6 to elements of the secretory pathway [11,47]. TMBIM proteins regulate Ca^2+^ homeostasis, possibly via a pH-dependent gating mechanism. This mechanism is particularly relevant within the secretory pathway (TMBIM4 in the GA; TMBIM6 in the ER), linking Ca^2+^ regulation to secretion and cellular pH [48]. In glioma cells, altered vesicle trafficking, characterised by reduced lysosomal degradation and a shift toward recycling endosomes, supports sustained secretion and membrane turnover, facilitating tumour growth and communication with neurons [49,50,51]. Concurrently, enhanced lysosomal function, partly driven by regulators such as palmitoyl-protein thioesterase 1 (PPT1), a regulator of lysosomal protein degradation, promotes autophagic flux and contributes to acidification of the tumour microenvironment [52,53]. Together, these coordinated alterations in lysosomal activity, vesicle dynamics, and autophagy can promote glioma invasiveness, angiogenesis, and therapeutic resistance [54].

Molecular function analysis of genes associated with TMBIM1/4 and TMBIM4/6 also highlighted lipase and phospholipase inhibitor activity, including Annexin A4 (ANXA4) and Annexin A5 (ANXA5), Ca^2+^-dependent phospholipid-binding proteins implicated in glioma proliferation, migration, invasion, and patient survival [55,56]. Another Ca^2+^-dependent phospholipid-binding protein, ANXA2, plays a crucial role in cell adhesion in glioma by facilitating the binding of tumour cells to the extracellular matrix [57]. Additionally, ANXA2 is stabilised by S100A11, activating NF-κB signalling, thereby establishing a positive feedback loop that enhances tumour proliferation, invasion, and stem-like properties [58]. The limited set of genes correlated with both TMBIM1 and TMBIM6 was insufficient to enable functional enrichment analysis. Although an overlap between genes correlated with TMBIM1 and TMBIM6 was identified, the number of shared genes was insufficient to yield statistically significant GO enrichment after multiple-testing correction. Consequently, no robust BP or MF categories could be assigned to this gene set.

In contrast, genes correlated with TMBIM3 and TMBIM5 were linked to pathways related to kinetochore organisation and chromosome condensation. Processes that are frequently dysregulated in glioma and are associated with chromosomal instability [59,60]. Consistently, cytoskeletal organisation and microtubule motor activity emerged as the most enriched molecular functions. The cytoskeleton, especially through tubulin-based microtubules, plays a key role in the organisation and segregation of chromosomes during cell division in glioma [61,62]. These microtubules assemble into kinetochore fibres that connect to kinetochores, enabling chromosome movement. This mechanism is critical for ensuring accurate chromosome segregation, a process that is frequently impaired in glioma cells [63,64].

Genes correlated with TMBIM2 and TMBIM3 showed only a modest enrichment; however, pathways related to cell cycle regulation and chromosome segregation were the most prominent for the TMBIM3/TMBIM5-associated gene set. Among the genes linked to cell cycle control, Aurora Kinase A (AURKA) integrates mitotic cell cycle regulation with Wnt/β-catenin-dependent transcriptional programmes, thereby enhancing proliferative capacity and stem-like self-renewal in glioma-initiating cells [65]. Similarly, Wee1-like protein kinase (WEE1), a key G2/M checkpoint kinase, is frequently overexpressed in high-grade gliomas and contributes to cell-cycle progression and therapy resistance [66,67]. These findings are consistent with the well-established role of chromosomal instability in glioma progression, particularly in glioblastoma, where widespread aneuploidy and recurrent copy number alterations are commonly observed [68,69]. Moreover, zinc ion transport is potentially associated with glioma progression [70].

Finally, genes correlated with TMBIM2 and TMBIM5 were enriched in synaptic vesicle maturation and plasticity. This observation was not surprising given the localisation of these proteins to the secretory pathway and plasma membrane [51,71,72,73]. Notably, although these biological processes partially overlap with those identified in the TMBIM4/TMBIM6 enrichment analysis, the associated gene sets differ substantially, indicating that TMBIM2 and TMBIM3 likely modulate similar functional programmes via distinct regulatory networks. Additionally, ER tubular organisation may be particularly relevant given the subcellular location of TMBIM2. In glioma, chronic ER stress promotes tubular dilation, paraptosis-like morphology, and adaptive survival mechanisms that contribute to chemoresistance and tumour progression [74,75].

Overall, the gene enrichment patterns observed reinforce the existence of two distinct TMBIM-associated transcriptional landscapes. The TMBIM1/4/6 group is linked to pathways involved in secretion and synaptic vesicle localisation, recycling, pH, autophagy, and lipase/phospholipase activity. In contrast, the TMBIM2/3/5 group is more associated with cell cycle regulation, chromosome segregation/instability and tubulin dynamics/motor processes and to a lesser extent with ER tubular organisation and synaptic vesicle recycling/maturation.

Further studies will be required to clarify the specific mechanisms through which TMBIM proteins 1–6 contribute to glioma biology and to determine which of these pathways are most relevant to tumour progression and therapeutic response.

## 4. Conclusions

This study reveals a previously unrecognised bipartite transcriptional organisation of the TMBIM family in glioma, characterised by opposing prognostic associations, grade-dependent expression patterns, and distinct transcriptional networks. Across independent datasets, TMBIM1, TMBIM4, and TMBIM6 were consistently associated with poorer survival and increased expression in higher tumour grades, whereas TMBIM2, TMBIM3, and TMBIM5 showed the opposite pattern. Expression correlation analyses further demonstrated that these genes segregate into two coherent transcriptomic patterns, a structure that is preserved across both LGG and GB.

Functional enrichment analyses supported this dichotomy, linking the TMBIM1/4/6 group to biological processes related to synaptic vesicle processing and lipase/phospholipase activity, while the TMBIM2/3/5 axis was associated with cell cycle regulation and tubulin cytoskeleton-associated processes. Together, these findings suggest that TMBIM family members participate in two distinct transcriptional programmes that most likely differentially influence or are influenced by glioma progression. A deeper understanding of the mechanisms underlying these TMBIM-associated regulatory networks may provide relevant insights into glioma biology and reveal potential prognostic markers and therapeutic targets. Importantly, data presented here suggest that the design of any future therapeutic approaches involving activity or expression manipulation of TMBIMs in glioma must be highly selective towards a specific group of TMBIMs.

## Data Availability

The datasets containing gene correlation analysis results (PCC values and gene names) and genes included in the Venn diagrams generated for this study can be found at https://doi.org/10.5281/zenodo.19226542.

## Supplementary Materials

The following supporting information can be downloaded at https://www.mdpi.com/article/10.3390/biology15141179/s1, Figure S1: Association between TMBIM gene expression and glioma subtype according to IDH status; Figure S2: Gene expression correlation analysis of TMBIM family gene expression in the Rembrandt dataset.
