# Supplementary material for: Transcriptomic Evidence Identifies Two TMBIM Subgroups with Opposing Prognostic Associations in Glioma
Source: Biology (Basel). 2026 Jul 17;15(14):1179. doi: 10.3390/biology15141179 (PMC13405653; doi:10.3390/biology15141179)
Supplement: Supplementary file 1 [file biology-15-01179-s001.zip › biology-4356668-supplementary.pdf]

Supplementary Figure S1 - Association between TMBIM gene expression and glioma subtype according to IDH status.

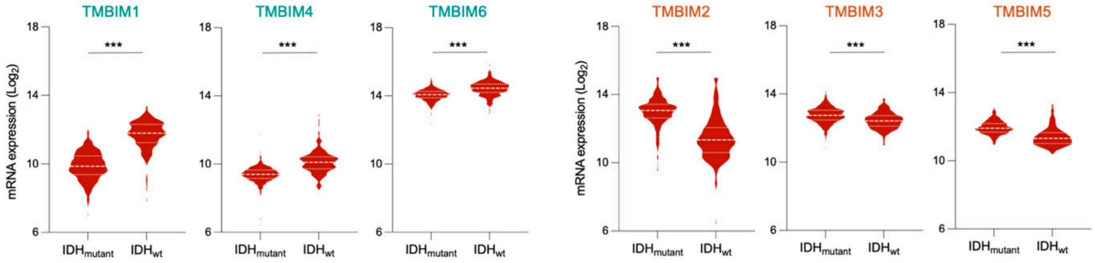

Figure S1. Association between TMBIM gene expression and glioma subtype according to IDH status. TMBIM1-6 gene expression in glioma according to IDH status (mutant or wild type (wt)). Group differences were assessed using one-way ANOVA followed by Tukey's HSD post hoc test for pairwise comparisons. *** $p < 0.001$.

Supplementary Figure S2 - Gene expression correlation analysis of TMBIM family gene expression in the Rembrandt dataset.

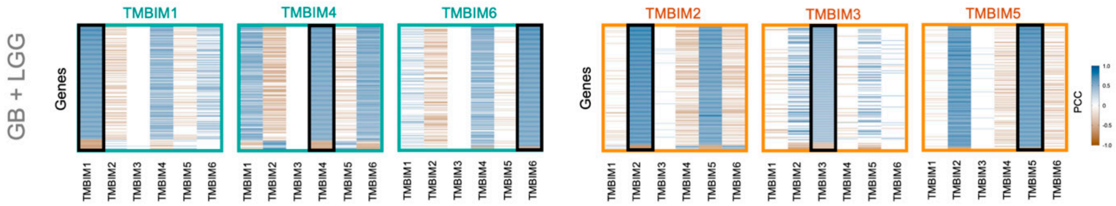

Figure S2. Gene expression correlation analysis of TMBIM family gene expression in the Rembrandt dataset. Heatmaps depict the top 300 genes most strongly correlated with each TMBIM family member, based on Pearson's correlation coefficient (PCC) calculated from gene expression data in the Rembrandt dataset. Blue gradient represents a positive Pearson's correlation, and brown gradient a negative correlation.
